# Hanging drop culture reprograms mesenchymal stem cell transcriptome to enhance cell delivery efficiency via attenuated pulmonary entrapment

**DOI:** 10.1007/s13346-025-01927-4

**Published:** 2025-09-06

**Authors:** Hsiang-Tzu Lee, Sheng-Yung Fu, Wei-Han Weng, Wei-Chen Chao, Yu-Pao Hsu, Nan-Ping Yang, Yu-Hsu Chen, Shau-Kwaun Chen, Chien-Wen Chang

**Affiliations:** 1 Department of Biomedical Engineering and Environmental Sciences, National Tsing Hua University, Hsinchu, 300044 Taiwan; 2 Department of Orthopedic Surgery, Taoyuan General Hospital, Ministry of Health and Welfare, Taoyuan, Taiwan; 3 Community Medicine Research Center, National Yang-Ming Chiao Tung University, Taipei, Taiwan; 4 Department of Orthopedics, Tri-Service General Hospital, National Defense Medical University, Taipei, Taiwan; 5 Department of Biology and Anatomy, National Defense Medical University, Taipei, Taiwan; 6 Institute of Neuroscience, National Chengchi University, Taipei, Taiwan

**Keywords:** Mesenchymal stem cell, Hanging drop method, Three-dimensional cell culture, Chemotaxis, Lung trapping

## Abstract

The three-dimensional (3D) culture system has emerged as an indispensable platform for modulating stem cell function in biomedicine, drug screening, and cell therapy. Despite a few studies confirming the functionality of 3D culture, the molecular factors underlying this process remain obscure. Here, we have utilized a hanging drop method to generate 3D spheroid-derived mesenchymal stem cells (3D MSCs) and compared them to conventionally 2D-cultured MSCs. The results showed that 3D MSCs exhibited distinct phenotypic features than 2D-cultured MSCs and expressed different transcriptional responses, as found from RNA-Seq analysis. Gene ontology (GO) annotations and KEGG pathway mapping pinpointed that the 3D MSCs responded more actively to incoming signals as they upregulated receptors and cytokine production while downregulating proteolysis-, cytoskeletal-, extracellular matrix-, and adhesion-related genes. Functionally, these MSCs also displayed enhanced chemotaxis and improved pulmonary transgression post-intravenous injection. This study provides mechanistic insights on addressing a significant limitation of MSC therapy: pulmonary entrapment after systemic delivery. Moreover, upregulated pluripotency-associated genes, such as Oct4, Sox2, and Nanog, suggest that the 3D MSCs possessed enhanced stemness and regenerative capacity. The results indicate that 3D spheroid culture reshapes MSC transcriptomic and functional profiles and emerges as a promising strategy for improving their therapeutic potential in regenerative medicine.

**Graphical abstract:**

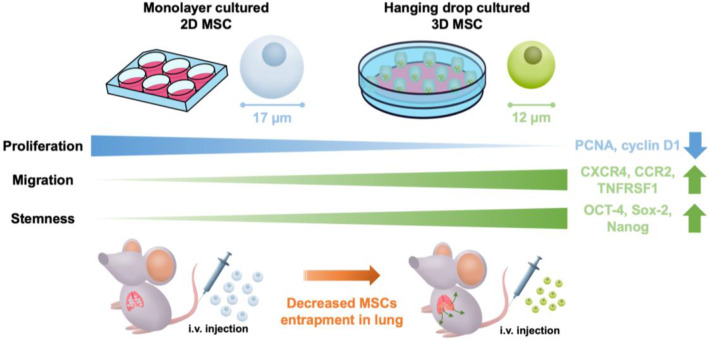

## Introduction

MSCs, have become a critical player in regenerative medicine and cell therapy, due to their ability of self-renewal and versatile potentials in clinical applications, such as neural repair, support of hematopoiesis, treatment of autoimmune disease, regeneration of bone, heart, liver, and kidney and so on [1, 2]. To be noticed, the phenotypes of MSCs, such as differentiation status and the varied secretome in different environmental factors, indicate that the therapeutic potentials for each specific application depend on particular cultural conditions [3, 4]. Thus, understanding and optimizing these the environmental factors, such as inflammatory, hypoxia, or metabolic environments, are crucial for enhancing the clinical applications of MSCs therapies. For example, MSCs could regulate in a counteractive manner based on the levels of inflammatory factors in their environment [5, 6]. The extrinsic factors that influence MSCs features could be the molecules released by neighboring cells, also known as paracrine, and from the circulation, cell-cell interaction, and cell-matrix interactions [4, 7]. The secreted factors, including cytokines and growth factors, and the adhesion molecules, such as I-CAM and V-CAM, interact with MSCs and regulate their proliferation and differentiation [8]. In fact, priming methods, such as preconditioning of MSCs under 3D culture conditions, have been reported to be necessary to switch those cells toward a more anti-inflammatory and pro-trophic phenotype and improve the therapeutic efficacies of MSCs [9–11]. Among various priming strategies, 3D culture has been suggested as a better culturing modality, which mimics native tissue environments and subsequently modulates MSCs possessing functional properties. Recent evidence has shown that 3D culture could provide an appropriate microenvironment for priming MSCs [12, 13].

Indeed, scientists have demonstrated the tremendous potential of 3D cell culture techniques in drug development and therapeutic applications [14, 15]. Compared to conventional 2D cell culture techniques, 3D culture provides spatial structures mimicking the tissue environments, allowing cell-cell interaction and cell-matrix interactions to maintain the native properties of the cells [16]. Given all these advantages, the 3D culture is considered a practical strategy for overcoming the limitations of 2D culture systems. Furthermore, 3D culture methods could be classified into two categories: scaffold-free and scaffold-based. A scaffold-free strategy can be applied for the cell types that exhibit self-aggregation properties, including MSCs, by inhibiting the cell attachment to the culture plate surface. In contrast, the cells grow on natural or synthetic materials to allow 3D cell organization in the scaffold-based systems [17]. Both methods have been applied to MSCs culture [18–20]. However, scaffold-based systems present notable technical challenges, including the difficulty of maintaining cellular characteristics during cell harvesting [21] and the latent interference with direct cell-cell or cell-extracellular matrix signal exchanges due to the presence of scaffold materials [22, 23]. Spheroid formation without a scaffold has become a widespread technique in culturing MSCs. Among the scaffold-free strategies, spheroids are one of the appealing options because of their enhancement of natural cell-cell interactions, which provides a more physiologically relevant microenvironment without exogenous materials.

The spheroid formation could be generated by cluster-based self-assembly (static formation) or collision-based assembly from cell suspensions (dynamic formation) methods [21, 24]. In dynamic formation methods, continuous stirring causes suspended cells to collide, facilitating their aggregation [24, 25]. In static formation methods, multicellular spheroids are formed with hanging drops, low-adhesion surfaces, or external forces such as gravitational force or magnetic field [24]. The Hanging Drop method suspends cells in culture medium droplets, using surface tension to form spheroids without requiring costly or complex equipment, making it easy and accessible. The spheroids allow direct cell-cell contact and interaction with the extracellular matrix (ECM), better mimicking the in vivo microenvironment for studying cell interactions [26]. The separated cells organize themselves into sections that facilitate interactions between cells, mirroring the natural processes seen in organ development. Significantly, the process of spheroid formation could affect the physical and biological properties of MSCs’ aggregates, which influence subsequent therapeutic efficacy. In the self-aggregated spheroids, the angiogenic, anti-inflammatory, and immunomodulatory features appeared to be promoted. Furthermore, the stemness and survival rates after transplantation were also enhanced [27]. Other research groups also demonstrated a series of phenotype alterations of MSCs when cultivated to form spheroid, such as decreased stiffness, modified cell surface antigen expression, and elevated CXCR4 expression (chemokine receptor that is involved in MSC homing in the body), and IL-24 (anti-cancer factors), modulating proliferation, paracrine effects and differentiation [18, 28, 29]. Recently, Lu and colleagues reported that the homogeneity of MSCs can be improved through 3D culture using the spinner flask method. Furthermore, the single-cell RNA sequencing (scRNA-seq) data revealed that MSCs polarized towards a more immunosuppressive phenotype, which effectively attenuated inflammation in a psoriasis animal model [30]. These observations have led to a growing interest in cultivating MSCs as multicellular spheroids within bioreactors, offering the possibility of easily scaling up to generate the large quantities of cells needed for therapeutic applications [24]. While those promising enhancements in functionality were exhibited, comprehensive insights into the molecular mechanism that underlies the described improvements remain limited. Furthermore, only a few direct comparison studies have evaluated their capabilities (2D vs. 3D MSC cultivation) in avoiding entrapment by the pulmonary vasculature in vivo.

To address these unresolved issues, we conducted a comparative investigation of 3D MSCs versus conventional 2D culture. We found that 3D MSCs cultured in the hanging drop were smaller and exhibited increased chemotaxis than 2D MSCs. These features resulted in reduced pulmonary entrapment of 3D MSCs following intravenous administration - an issue that currently threatens the full potential of MSC therapies [31]. Taken together, our results are a strong argument for the 3D spheroid culture as a potent means for modulating transcriptomic profiles and inducing a series of functional changes, such as increased cellular responses to immune stimuli, decreased cell-cell and cell-matrix adhesion, promoted chemotaxis ability and enhanced in vitro stemness and functionally to enhance their translational prospects.

## Materials and methods

### Cell culture

Human primary Wharton’s Jelly mesenchymal stem cells (hMSCs) were obtained from the Bioresource Collection and Research Center (BCRC, Taiwan). In this study, hMSCs were cultured with 5% CO_2_ at 37 °C in 80% Minimum essential medium (Eagle) with Earle’s BSS, 2 mM L-glutamine, 1.5 g/L sodium bicarbonate, 0.1 mM non-essential amino acids, 1.0 mM sodium pyruvate, 20% fetal bovine serum (FBS), 4 ng/mL Human-bFGF, and 100 U/mL Penicillin/Streptomycin (P/S). MDA-MB-231, human triple-negative breast cancer cells, were cultured in Dulbecco’s Modified Eagle’s Medium (DMEM, Gibco; Thermo Fisher Scientific, Inc.) containing 10% FBS and 1% P/S. Upon reaching a density of 90%, the cell culture vessel was replaced at a ratio of 1:3 and placed in a 37 °C and 5% CO₂ incubator for further culture.

### Hanging drop 3D culture

The present experiment employs hanging drop culture to facilitate the formation of cell clusters, with the objective of reducing cell size for subsequent therapeutic applications. A total of 2 × 10⁴ cells/ 20 µL should be added to the upper surface of a 10 cm culture dish. Subsequently, 5 mL of 1X PBS should be added to the lower surface to ensure the cells have sufficient water for continuous growth. This is important to prevent the cells in the small-volume culture from lacking water due to the 37 °C control in the cell culture incubator.

### Cell size measurement

The cultured 24-, 48-, or 72-hour single hMSC cell clusters should be placed into a 48-well plate for microscopic observation and photography. To disperse the cells, pipette 25 pieces of the hMSC cell clusters into a 1.5 mL tube and centrifuge at 1500 rpm for 5 min. A total of 250 mL of 1X PBS should be added for a second wash, after which the supernatant should be removed, and 250 µL of 0.25% Trypsin-EDTA/ collagenase/ hyaluronidase cell interstitial enzyme action should be added for 15 min. Subsequently, 250 mL of 20% FBS α-MEM should be added to terminate the Trypsin reaction. The solution is filtered through a 40-µm cell strainer to remove larger clumps or other debris. Subsequently, centrifugation at 3000 rpm for 10 min is performed to collect the cells, after which the supernatant is removed, and the cells are resuspended in 100 µL 20% FBS α-MEM culture medium. Finally, the cell size and viability are measured.

A 10 µL sample should be mixed with Trypan blue at a 1:1 ratio in order to ascertain the dimensions, viability, and quantity of cells utilizing a cell counter (Countess 3, Thermo Fisher Scientific, Inc). The cell recovery rate can then be calculated using the obtained cell number.

The cell recovery rate is calculated as follows:

$$\eqalign{& {\rm{Cell}}\,{\rm{recovery}}\,{\rm{rate}}\left( {\rm{\% }} \right)\,{\rm{ = Number}}\,{\rm{of}}\,{\rm{recovered}} \cr & {\rm{cell/}}\left( {{\rm{Number}}\,{\rm{of}}\,{\rm{starting}}\,{\rm{cells}}\,{\rm{ \times Number}}\,{\rm{of}}\,{\rm{cell}}\,{\rm{clusters}}} \right){\rm{ \times 100}} \cr} $$

The cells should be suspended in ice-cold media and sampled at 0, 30, 60, 90, 120, and 180 min. At each time point, 10 µL of the suspended cells should be mixed 1:1 with Trypan blue in order to record the cell size, cell viability, and stability of the cells over time using a cell counter.

### Chemotaxis assay

A total of 4 × 10⁴ cells/well of the MDA-MB-231 cells were placed at the bottom of a 24-well plate. The culture medium was then added for a period of two days, during which time the orientation of both the 2D and 3D cultured hMSCs was analyzed. The MDA-MB-231 cells were washed once with 1X PBS and replaced with culture medium (600 µL, 10% FBS DMEM). The non-tumor cell group was added with 600 µL 10% FBS DMEM. A 24-well plate was placed within a Transwell apparatus, and the 2D and 3D cultured hMSCs were resuspended in 200 µL of serum-free DMEM and added to the Transwell upper layer. A comparison of the 2D and 3D cultured hMSCs without tumor cells and with MDA-MB-231 cells-induced groups, respectively, is required. The culture was maintained for 24 h, after which the 1X PBS was used to wash the apparatus twice. Subsequently, 600 µL of 4% PFA was added to the lower layer of the Transwell apparatus to fix the cells, while 200 µL of the same solution was added to the upper layer of the apparatus for a 10-minute fixation period. Following this, the apparatus was rinsed once with 1X PBS. The unmigrated hMSCs cells in the upper layer of the Transwell should be scraped off with a cotton swab, after which the upper layer should be rinsed twice with 1X PBS. Subsequently, 600 µL of 4 µg/mL Hoechst should be added to the lower layer of the Transwell to stain it for 15 min, after which the lower layer should also be rinsed twice with 1X PBS.

### Tracing MSC in vivo

The luciferase-expressing stem cells (^luc^hMSC) were constructed by lentivirus transduction according to the protocol provided from RNAi core facility (Academia Sinica, Taipei, Taiwan). Six- to eight-week-old NOD/SCID mice were anesthetized with inhaled anesthetics (2 L/min O_2_ and a 4% isoflurane mixture) and intravenously injected via the tail vein with 1 × 10^6^ 2D and 3D cultured ^luc^hMSC suspended in 200 µL of PBS. Following the injection, the IVIS was employed at fixed time points (3, 24, 48, and 120 h) to observe the biodistribution of the cells in the mouse. Ten minutes prior to IVIS detection, 6 mg of D-luciferin was dissolved in 250 µL of PBS and injected intraperitoneally. Detection was initiated after the animal was anesthetized with gas.

### RNA preparation

Total RNA was prepared in accordance with the instructions provided in the RNAzol^®^ RT (RN190, MRC, Molecular Research Center, Inc., USA). The following conditions should be observed: 0.5 mL of RNAzol^®^ RT should be added to the cells to homogenize, followed by the addition of 0.2 mL of water to facilitate thorough mixing. After a 15-minute waiting period, centrifugation at 12,000 xg for 15 min should be performed. The supernatant should then be aspirated and an equal volume of isopropyl alcohol added and mixed evenly. After a 15-minute waiting period, the solution should be centrifuged at 12,000 xg for 10 min. Subsequently, 75% alcohol should be added and the solution centrifuged at 4,000 xg for 10 min. This process should be repeated twice. The solution should then be washed twice. Finally, the solution should be blown dry, redissolved in water, and stored in a refrigerator at -80 °C. The purity and quantity of the RNA were determined using a SimpliNano™ Biochrom spectrophotometer (Biochrom, MA, USA). The integrity and degradation of the RNA were monitored using a Qsep 100 DNA/RNA Analyzer (BiOptic Inc., Taiwan).

### Library preparation and sequencing

For the preparation of RNA samples, 1 µg of total RNA was used as the starting material. The sequencing libraries were created using the KAPA mRNA HyperPrep Kit (KAPA Biosystems, Roche, Basel, Switzerland), following the guidelines provided by the manufacturer. Index codes were subsequently added to assign sequences to each sample. In brief, mRNA was extracted from the total RNA using magnetic oligo-dT beads. The isolated mRNA was then fragmented by heating it at an elevated temperature in the presence of magnesium within the KAPA Fragment, Prime, and Elute Buffer (1x). First-strand cDNA was generated using random hexamer primers. The subsequent combined process of second strand synthesis and A-tailing transforms the cDNA: RNA hybrid into double-stranded cDNA (dsDNA), incorporates dUTP into the second cDNA strand, and attaches dAMP to the 3’ ends of the resulting dsDNA. The dsDNA adapter, featuring 3’ dTMP overhangs, was then ligated to the library insert fragments, creating library fragments with adapters. For optimal selection of cDNA fragments measuring between 300 bp and 400 bp in length, the library fragments were purified using the KAPA Pure Beads System (KAPA Biosystems, Roche, Basel, Switzerland). The library, having been modified with the adapter sequences on either end suitable to the library, was further amplified using the KAPA HiFi HotStart Ready Mix (KAPA Biosystems, Roche, Switzerland) with the Library Amplification Primers. The amplification left out the strand-bearing dUTP, thereby allowing for strand-specific sequencing. The PCR products were purified with KAPA Pure Beads, and the quality of the library was evaluated with the Qsep100 DNA/RNA Analyzer (BiOptic Inc., Taiwan). The Qubit^®^ 2.0 Fluorometer (Thermo Scientific) and the Agilent Bioanalyzer 2100 system were used for quality assessment of the library. Finally, the library was sequenced in the Illumina NovaSeq 6000 platform, enabling 150 bp paired-end reads at TOOLS (Biotools, Taiwan).

### MTT assay

Cell viability was evaluated via colorimetric MTT assay. A total of 5,000–10,000 cells were seeded in each well of the 96-well plates. Following a 24-hour incubation period, the medium was replaced with fresh medium, and the plate was incubated for an additional 24, 48, and 72 h. Subsequently, 20 µL of 5 mg/mL MTT was added to each well and incubated for 2 h at 37 °C. The formazan crystals were then dissolved with 100 µL of Dimethyl Sulfoxide (Thermo). Each assay will be conducted in triplicate, and all experiments will be repeated at least three times. A purple-colored solution was obtained. Optical density (OD) of the formazan solution was estimated by measuring the absorbance at 570 nm using a microplate reader. The cell viability percentage will be estimated using the following formula:

Cell viability (%) = absorption test/absorption control × 100%.

### Reverse transcription quantitative polymerase chain reaction (RT-qPCR) assay

Reverse transcription was performed using the ToolsQuant II Fast RT Kit (Tools, Taiwan) in accordance with the manufacturer’s instructions. The reaction was incubated at 42 °C for 3 min, followed by the removal of gDNA and subsequent storage on ice. The gDNA removal and reverse transcription reactions were thoroughly mixed and incubated at 42 °C for 15 min. Each reaction was terminated at 95 °C for 3 min and then placed on ice. Quantitative reverse transcription-polymerase chain reaction (RT-qPCR) was conducted using the PowerUp SYBR Green Master Mix (Thermo Fisher Scientific) in accordance with the manufacturer’s instructions. This experiment was conducted in triplicate. A real-time RT-qPCR was conducted on a Bio-Rad CFX Connect with SYBR Green under the following conditions: The reaction was incubated for two minutes at 50 °C, two minutes at 95 °C, and then subjected to a three-step cycle comprising 20 s at 90 °C, 20 s at 57 °C, and 30 s at 72 °C for 40 cycles, followed by an additional dissociation curve. The specific primers utilized in RT-qPCR were listed in Table 1.

**Table 1:** The information of primers sequence in RT-qPCR

Name	Sequence
hSOX2-F	GCTACAGCATGATGCAGGACCA
hSOX2-R	TCTGCGAGCTGGTCATGGAGTT
OCT-4-F	CTTGCTGCAGAAGTGGGTGGAGGAA
OCT-4-R	CTGCAGTGTGGGTTTCGGGCA
Nanog-F	AATACCTCAGCCTCCAGCAGATG
Nanog-R	TGCGTCACACCATTGCTATTCTTC
Ki-67-F	TTCGCAAGCGCATAACCCA
Ki-67-R	AACCGTGTCACAGTGCCAAA
PCNA-F	CCTGCTGGGATATTAGCTCCA
PCNA-R	CAGCGGTAGGTGTCGAAGC
Cyclin D1-F	GCTGCGAAGTGGAAACCATC
Cyclin D1-R	CCTCCTTCTGCACACATTTGAA
CXCR4-F	CTCCTCTTTGTCATCACGCTTCC
CXCR4-R	GGATGAGGACACTGCTGTAGAG
CCR2-F	TGCAAAAAGCTGAAGTGCTTG
CCR2-R	CAGCAGAGTGAGCCCACAAT
TNFRSF1b-F	TTCATCCACGGATATTTGCAGG
TNFRSF1b-R	GCTGGGGTAAGTGTACTGCC
GAPDH-F	AGCCACATCGCTCAGACAC
GAPDH-R	GCCCAATACGACCAAATCC

### Statistical analysis

The data were presented as the pooled mean ± standard error of the mean from a minimum of three independent experiments. Statistical analysis was performed using GraphPad Prism 10 software. (GraphPad Software, Inc., La Jolla, CA, USA). To compare the data from the control and treatment groups, multiple or Student’s t-tests were employed. The symbols were considered to indicate a statistically significant difference at the following levels: *P < 0.05, **P < 0.01, ***P < 0.001, and ****P < 0.0001.

## Results

### The size of MSCs was decreased in the hanging drop culturing system

**Fig. 1:**
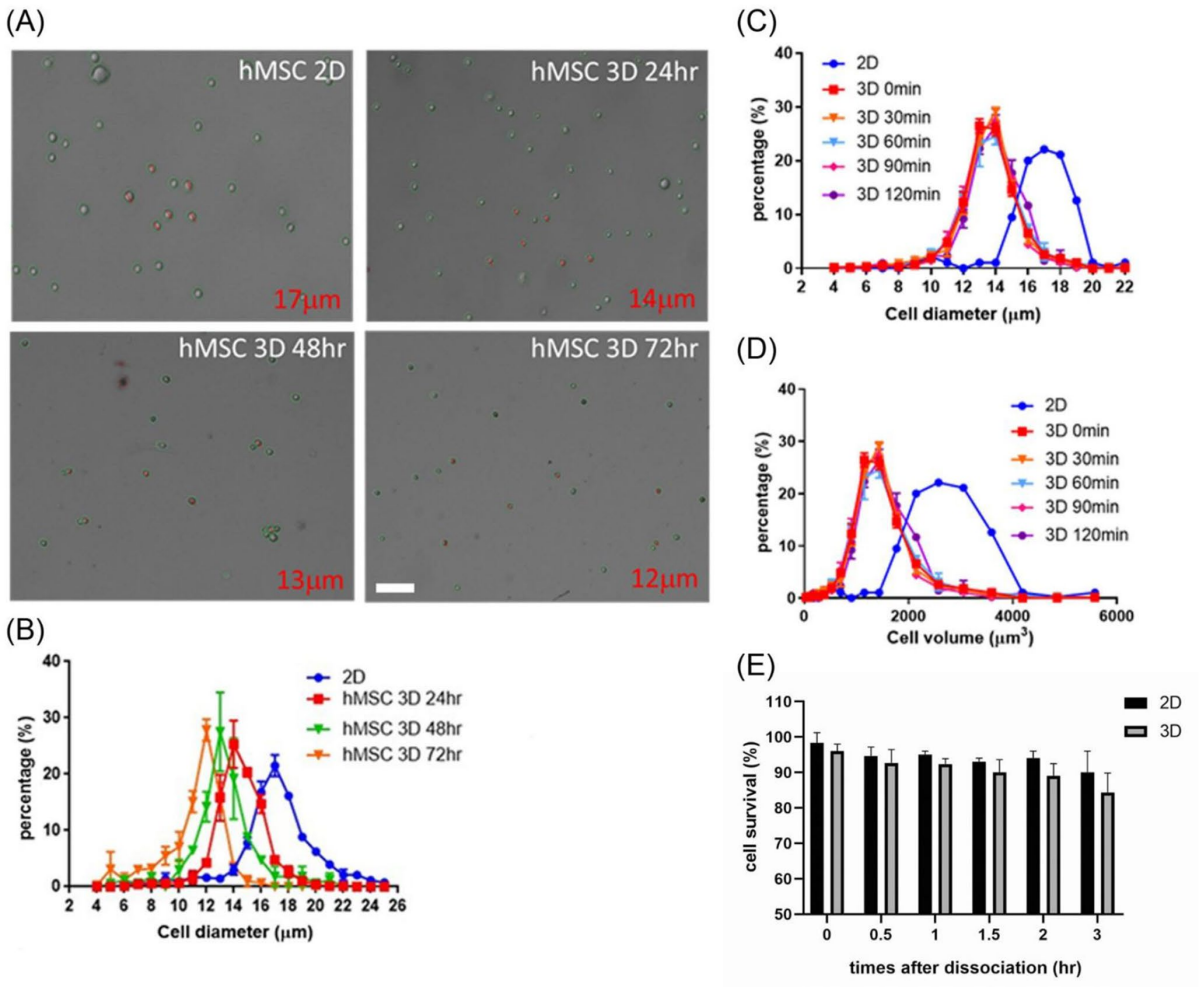
MSC cell size was decreased in the hanging drop culture. A comparative analysis of the cell diameter changes of 2D and 3D cultured human mesenchymal stem cells was performed at different culture periods. (**A**) The cell counter displayed the cell morphology. (bar = 200 μm) (**B**) The cell counter calculated the cell diameter results. This study aimed to test and quantify the diameter and volume changes of 2D and 3D cultured human mesenchymal stem cells within one hour after being broken up. Figures (**C**) and (**D**) illustrate these cells’ diameter and volume change. (**E**) The quantitative data depicts the outcome of the Trypan Blue test, indicating the cell activity. The experiment was conducted with three replicates (*n* = 3)

Human Wharton’s Jelly mesenchymal stem cells (hMSCs) were employed in this study to investigate the phenotypic differences between 3D and 2D culturing modality. Several basic features of the cells, such as cell morphology, size and cell survival were first analyzed. hMSCs were cultured in hanging drop for 24, 48, and 72 h. Then the 3D spheroids were dissociated for cell imaging and cell diameter measurement. Morphological evaluation revealed that 3D culture did not induce noticeable detrimental cellular effects such as membrane blebbing or swelling (Fig. 1A). Both cell images and the cell diameter measurement revealed decreased cell size of hMSCs in hanging drop compared with those in 2D culture (Fig. 1B). In 2D culture, the average diameter of hMSCs was about 17 μm, while hMSCs cultured in hanging drops for 24, 48, and 72 h exhibited average diameters of 14, 13, and 12 μm, respectively. As the culture time increases, the cell size decreases accordingly. The cell diameter and volume, furthermore, were relatively stable for at least 2 h (Figs. 1C&D), indicating that the size differences are probably due to intrinsic cellular events rather than transient mechanical compression resulting from spheroid aggregation during the immediate time of culture. The dissociated cells were stained with Trypan Blue to evaluate their survival. The quantitative data demonstrated a comparable survival rate between MSCs undergoing 2D and 3D culture in consecutive monitoring (Fig. 1E, all > 80%). These findings indicate that 3D spheroid culture significantly decreases cell size without compromising morphology or viability. This phenotypic alteration forms the basis for investigating whether morphological changes correlate with transcriptomic shifts and functional enhancements.

### Differential expression of genes between MSC in 2D and 3D culture

**Fig. 2:**
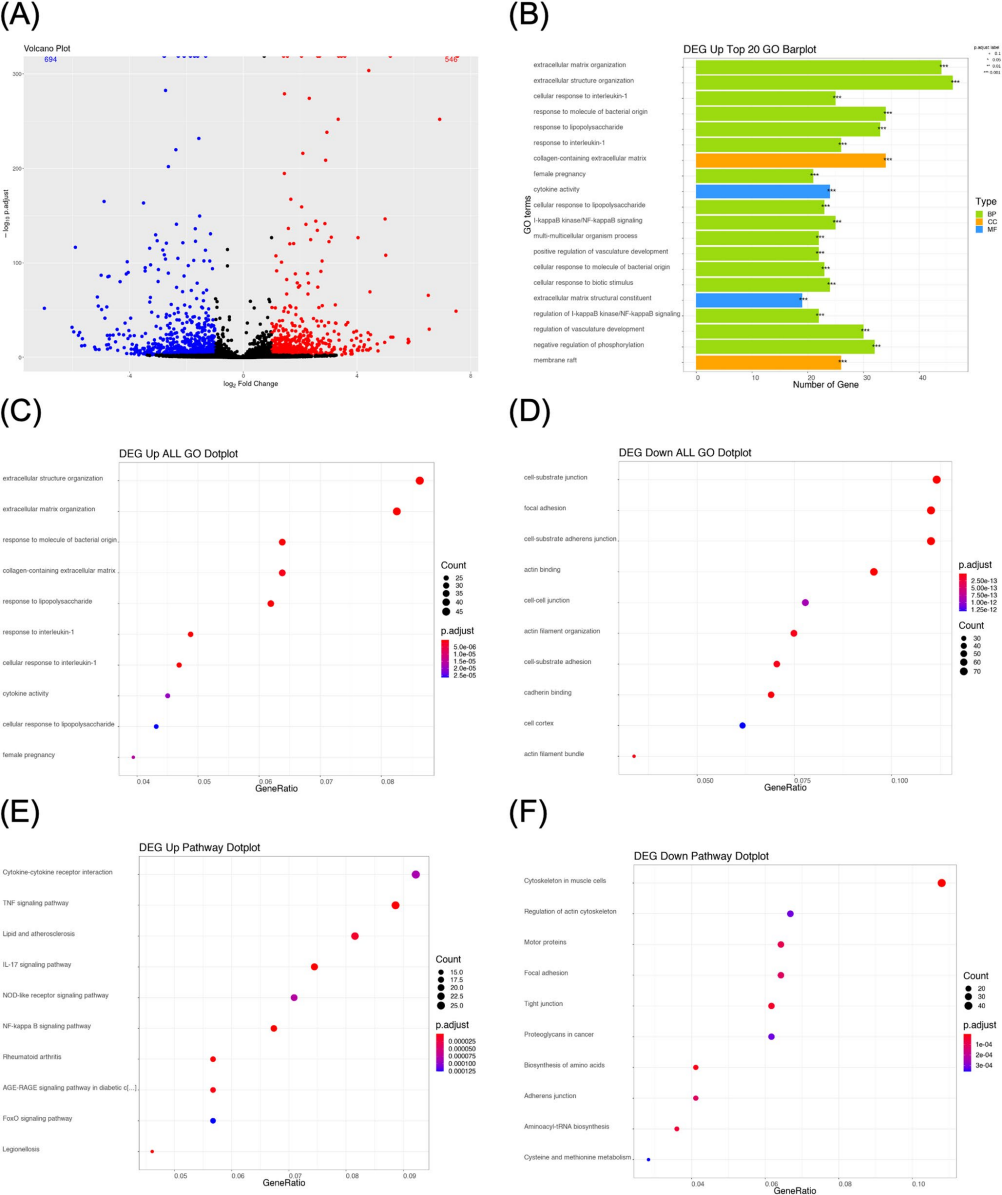
Differential expression of genes between MSC in 2D and 3D culture. (**A**) The volcano plot shows the DEGs between the 2D and 3D cultured mesenchymal stem cells. Each point symbolizes a gene. Red and blue points signify upregulated and downregulated DEGs, respectively, while black points represent genes that are not differentially expressed. (**B**) GO enrichment analysis shows that the terms are significantly enriched with the identified DEGs between 2D and 3D cultured mesenchymal stem cells. (**C**) The top 10 Up-regulation and (**D**) Down-regulation GO enrichment analysis in the 2D and 3D cultured mesenchymal stem cells. (**E**) The top 10 Up-regulation KEGG pathways. The x-axis indicates the generation of the enriched genes, while the y-axis lists the names of the enriched pathways. The size of each dot corresponds to the number of enriched genes, and the color signifies the significance of the p.adjust value, with red denoting high significance and blue indicating low significance. (**F**) The top 10 Down-regulation KEGG pathways. The x-axis indicates the generation of the enriched genes, while the y-axis displays the names of the enriched pathways. The size of each dot corresponds to the number of enriched genes, and the color signifies the significance of the p.adjust value, with red denoting high significance and blue indicating low significance

Since the above results suggested phenotypic changes under 3D culture, we next performed a comparative transcriptomic analysis to further characterize the molecular alterations. hMSCs were cultured in a hanging drop for 48 h, and the multicellular aggregates were directly lysed without matrix dissociation to obtain total RNA. RNA samples collected from 2D or 3D cultured MSCs were analyzed by RNA sequencing. The comparative transcriptomes identified differential expressed genes (DEGs), which were further compared by ontology and KEGG assays. A total of 1,240 genes were identified as significantly differentially expressed (FC > 1, P.adjust < 0.005) between the 2D and 3D MSCs. In 3D MSCs, a total of 546 genes showed increased expression, while 694 genes exhibited decreased expression (Fig. 2A). We conducted an over-representation analysis using the Gene Ontology (GO) databases to assess the biological importance of DEGs, which was used for classification and functional annotation. The top 20 GO categories were enriched for 16 Biological Processes (BP), including the responses to immune stimuli such as lipopolysaccharides and interleukin-1, NF-κB signaling extracellular organization, positive regulation of vasculature development, and cellular responses to a biotic stimulus; 2 Cellular Component (CC), including collagen-containing extracellular matrix and membrane raft; and 2 Molecular Function (MF), cytokine activity and extracellular matrix constituents (Fig. 2B). From the analysis (Figs. 2C&D), it was found that the predominant upregulated GO processes were associated with cellular responses to immune stimuli and extracellular organization, while downregulated processes pertained to cell-substrate adhesion, cell-cell junction formation, focal adhesion, actin binding and organization, and cadherin binding.

KEGG pathway enrichment analysis was conducted to gain further insights into the functional classification and pathway assignments of the DEGs. Among the upregulated pathways, the top 10 pathways included cytokine-cytokine receptor interaction pathway, TNF signaling, lipid and atherosclerosis, IL-17 signaling, NOD-like receptor signaling, NF-kappa B signaling, Rheumatoid arthritis, AGE-RAGE signaling, FoxO signaling and legionellosis pathway (Fig. 2E). Conversely, the top 10 downregulated pathways included regulation of actin cytoskeleton, motor proteins, focal adhesion, tight junction, proteoglycans in cancer, biosynthesis of amino acids, adheren junction, aminoacyl-tRNA biosynthesis, and cysteine and methionine metabolism (Fig. 2F). These results are consistent with the GO enrichment analysis, further indicating that 3D spheroid culture promoted inflammatory and immune-related signaling while attenuating cytoskeletal structure and cell adhesion functions. Overall, these transcriptomic transitions indicate that 3D spheroid culture may govern the immuno-responsiveness of MSCs along with the vascular-related pathways and cytoskeletal dynamics. Therefore, we next examine whether such molecular changes lead to functional changes, especially regarding chemotactic behavior and tissue trapping after systemic delivery.

### Chemotaxis properties have been enhanced by 3D culture

**Fig. 3:**
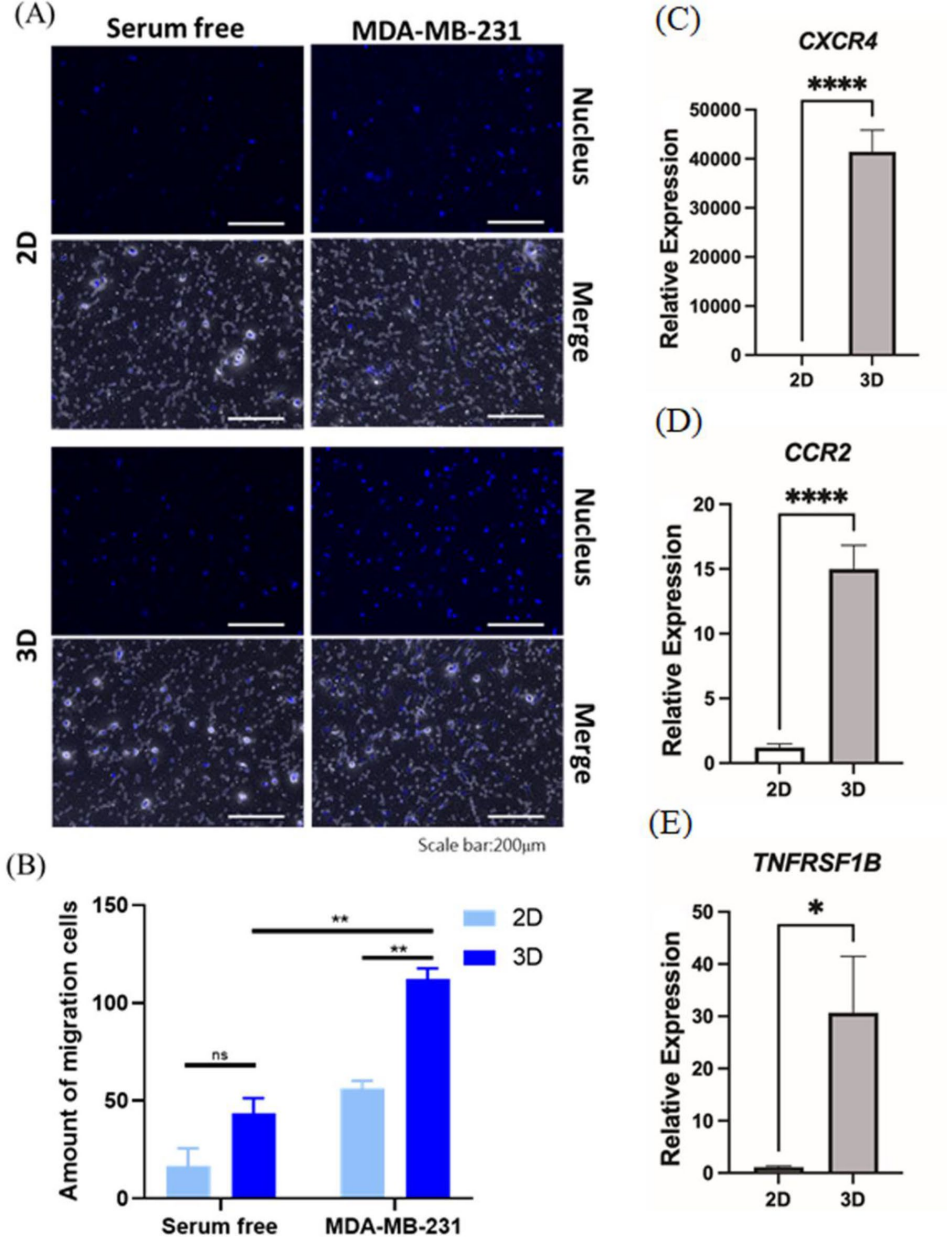
Chemotaxis properties have been enhanced by 3D culture. The migration ability of stem cells cultured in 2D and 3D under the induction of triple-negative tumor cells. (**A**) The MSC cells were cultured in a serum-free environment for 48 h, and the MDA-MB-231 cell induction environment was observed using a fluorescence microscope. The fluorescent images of migrating cells were captured with a scale bar of 200 microns. (**B**) The number of fluorescent particles was calculated using the ImageJ software function (*n* = 3, Each column represents the mean ± standard error of the mean of at three independent experiments. **p < 0.01, ns non-significant.). The elevation of chemotaxis ability could result from increasing expression of chemoattractant cytokine receptors. The expression of chemokine-receptor genes (**C**) *CXCR4*, (**D**) *CCR2*, and (**E**) *TNFRSF1B* were determined by RT-qPCR (*n* = 3, Each column represents the mean ± standard error of the mean of at three independent experiments. *p < 0.05, ****p < 0.0001)

Both GO and KEGG results suggested the promotion of chemotaxis abilities of MSCs after hanging drop culture. The chemotaxis assay was conducted to determine the migration of MSCs toward cancer cells. MDA-MB-231 is a cancer cell line derived from a triple-negative breast cancer patient and was applied as a chemoattractant source of tumor. The MSC culture alone, without the presence of cancer cells, was applied as the negative control. Only a few MSCs crossed the transwell without cancer cells, and no significant difference existed between cells from 2D or 3D cultures (Figs. 3A&B). However, when MDA-MB-231 cells were co-cultured with cancer cells, 3D MSCs exhibited a significantly enhanced chemotactic response compared to 2D MSCs. These results implied that the 3D culture enhanced the chemotaxis ability, not the mobility of MSCs. To investigate the molecular basis behind this enhanced chemotaxis, we profiled the expression of certain molecular markers associated with MSC homing. SDF1-CXCR4 and CCL2-CCR2 are the well-known axis of chemokines and receptors that could mediate the recruitment of MSCs into tumors [31]. Transcriptomic analysis revealed upregulated expression of Cxcr4, Ccr2, and Tnfrsf1b (the gene that encodes another receptor associated with chemotaxis, TNFRSF1B) in 3D MSCs. These changes were subsequently validated by real-time PCR (Figs. 3C-E). The upregulation of CXCR4, CCR2, and TNFRSF1B supports the hypothesis that 3D culture conditions skew MSCs for enhanced chemotaxis toward tumor-derived chemo-attractants.

### 3D culture method prevents lung entrapment of the intravenously-injected MSC

**Fig. 4:**
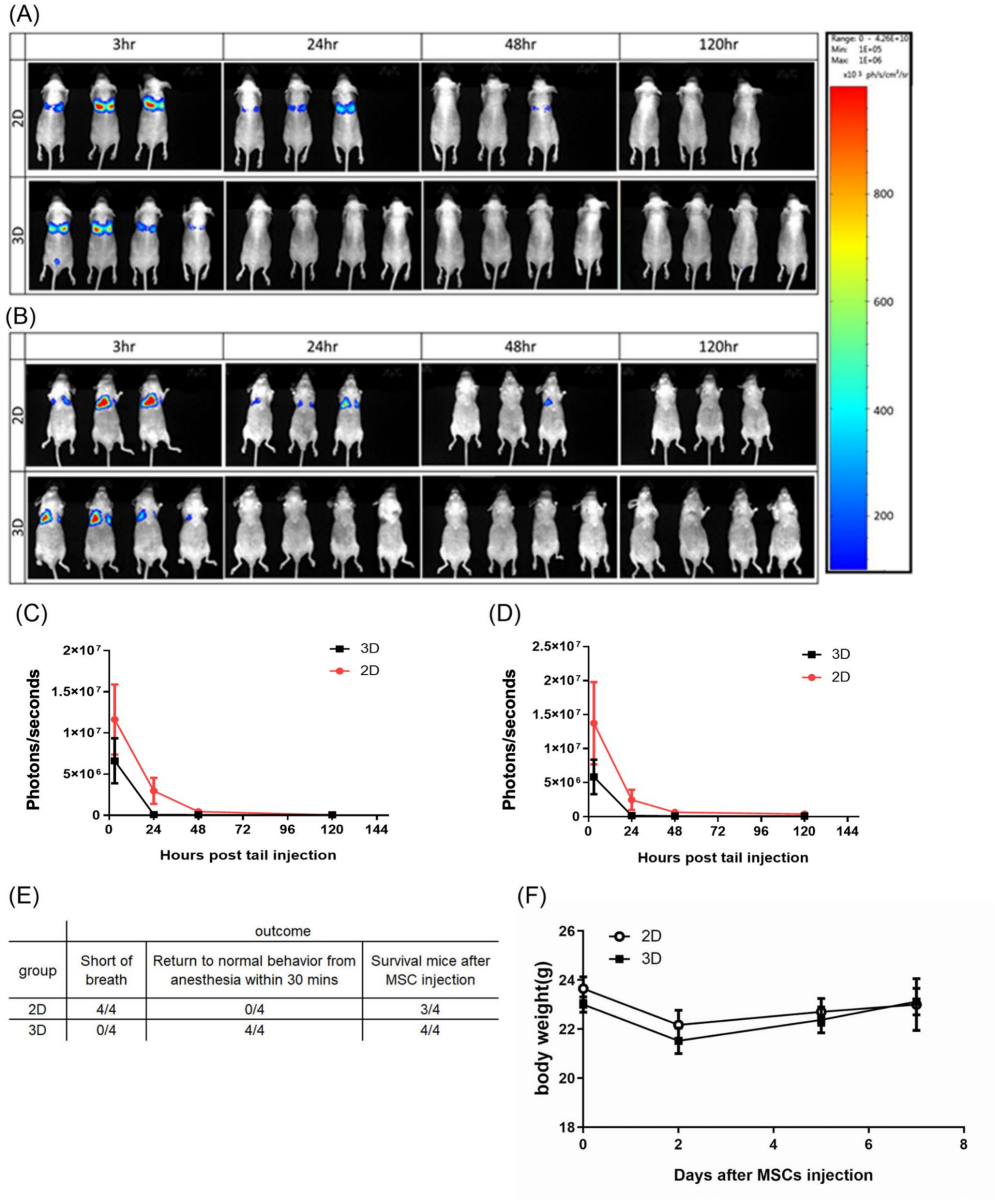
Hanging drop culture method prevent tissue trapping when the MSC is intravenously administrated. This study aimed to examine the effects of 2D and 3D hanging drops cultured to ^Luci^hMSCs stem cells in mice. The results are presented in quantitative form. (**A**) Back image. (**B**) Abdominal image. (**C**) Back signal quantitative analysis. (**D**) Abdominal signal quantitative analysis. (**E**) Physiological status monitoring of experimental mice after hMSCs injection (**F**) The body weight record for experimental mice after injection of hMSCs (N ≧ 3 for each group)

According to earlier studies, one of the most important factors in rejuvenating the therapy through intravenous delivery of stem cells was the prominent retention of infused cells in the pulmonary circulation [32]. Since 3D culturing could decrease the size of MSCs, as well as the cell adhesion capability, which implied that the MSCs could avoid being trapped in the lung and improve their circulation time. Therefore, we conducted an in vivo tracking study using hMSCs that expressed the luciferase reporter gene (^Luc^hMCSs). The ^Luc^hMCSs were subjected to either conventional 2D or 3D spheroid culture. The hMSCs were dissociated into single cells and injected intravenously via tail vein into the nude mice. The IVIS images (Figs. 4A&B) and its quantitative data (Figs. 4C&D) revealed that 2D MSCs showed stronger and longer-lasting lung accumulation than 3D counterparts. Moreover, the physiological status and body weight monitoring of experimental mice after hMSC injection are shown in Fig. 4E and F, respectively. This indicates that the injection of larger-sized hMSCs cultured in 2D condition affects the respiratory status of the mice. The loss of respiratory distress signs in mice injected with 2D MSCs was attributed to the considerable pulmonary accumulation of the injected cells. In contrast, the retention in the lungs was limited for the 3D MSCs. In addition, 3D culturing altered the distribution dynamics of MSCs invasion by bioluminescence imaging in 2 out of 6 mice. This data indicated that the reduced cell size and decreased expression of genes related to cell adhesion by 3D culture resulted in a significant reduction of lung entrapment.

### The 3D culture method decelerated MSC proliferation

**Fig. 5:**
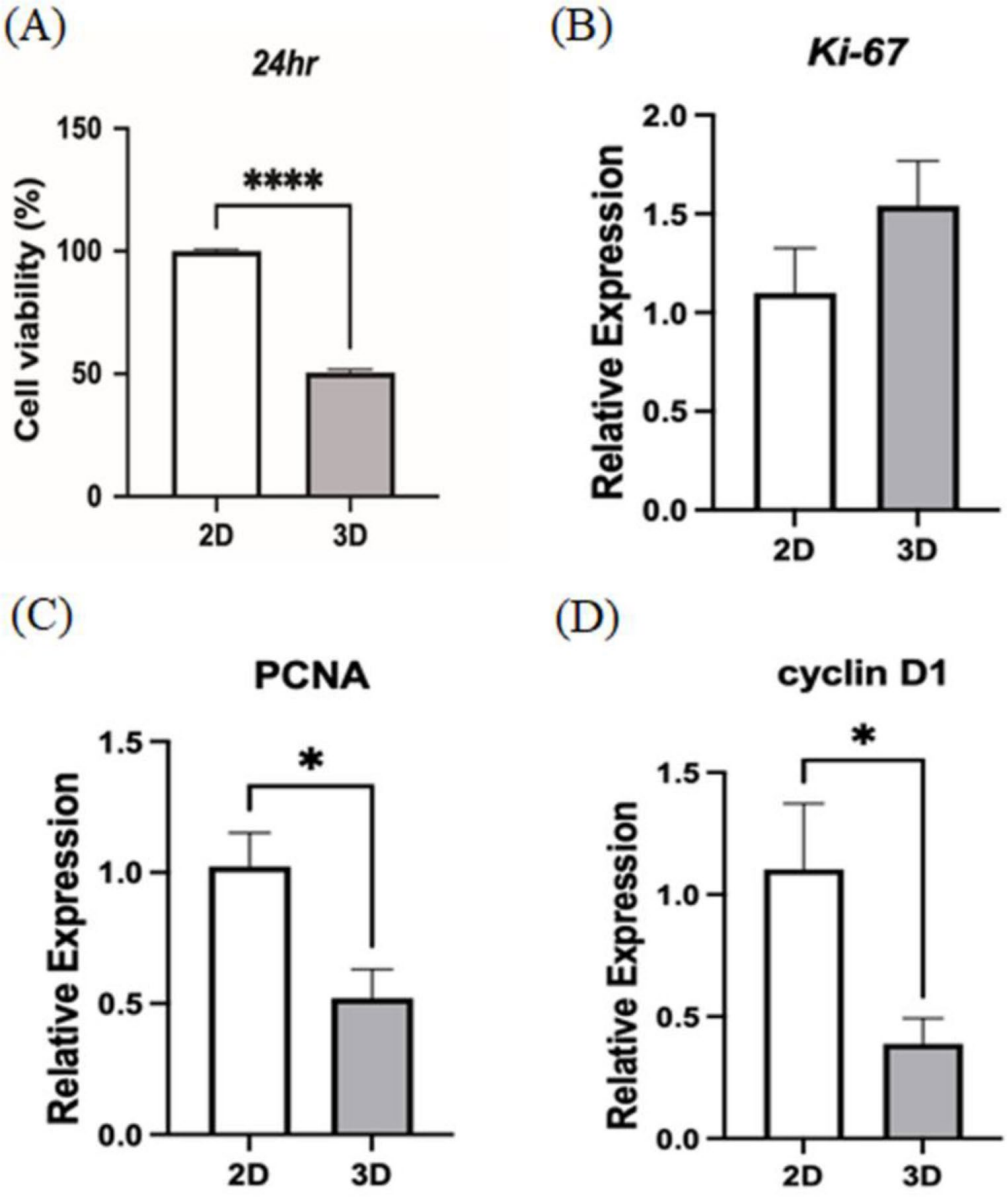
The hanging drop culture method decelerated MSC proliferation. (**A**) The effect of hanging drop culture method on cell viability in MSCs for 24 h. Each bar represents the mean ± standard error of the mean of at three independent experiments. ****p < 0.0001. To ascertain whether the hanging drop culture method influenced the cell cycle of the MSCs, the expression of cell-cycle relevant genes, namely (**B**) Ki67, (**C**) PCNA, and (**D**) cyclin D1, was determined by RT-qPCR. (*n* = 3, Each column represents the mean ± standard error of the mean of at three independent experiments. *p < 0.05)

From previous RNA-seq analysis, MSCs in 3D culture showed many differences in gene expression compared to those in 2D culture, particularly in the expression of adhesion proteins and related cytoskeleton. The downstream gene expression influenced by these adhesion proteins affects the survival ability of MSCs. Therefore, we subsequently investigate whether 3D culture impacts the replicative capacity of MSCs. MSCs were cultured in hanging drop and treated with trypsin and collagenase to dissociate the cells. MSCs isolated from 2D or 3D culture were separately plated for 24 h of incubation to calculate the cell proliferation by MTT assay. Starting from the same cell number, the 2D MSCs outscored those from 3D culture by nearly two times (Fig. 5A). Further analysis was carried out on the expression of selected cell cycle genes to validate the findings above. Ki67, a marker generally expressed throughout all active phases of the cell cycle (G1, S, G2, and M phases) [33], was maintained at high levels in both groups. However, the expression of the proliferating cell nuclear antigen (PCNA) associated with DNA replication during S-phase [34], and that of the Cyclin D1, which regulates G1/S transition [35], were significantly downregulated in 3D MSCs (Figs. 5B-D). This continuous expression of Ki67, but reduced PCNA and Cyclin D1, suggested that 3D culture might induce a quiescent-like state of MSCs, where cells remain arrested in interphase without actively progressing through DNA synthesis and division.

### The pluripotent genes were upregulated in MSC in 3D culture

**Fig. 6:**
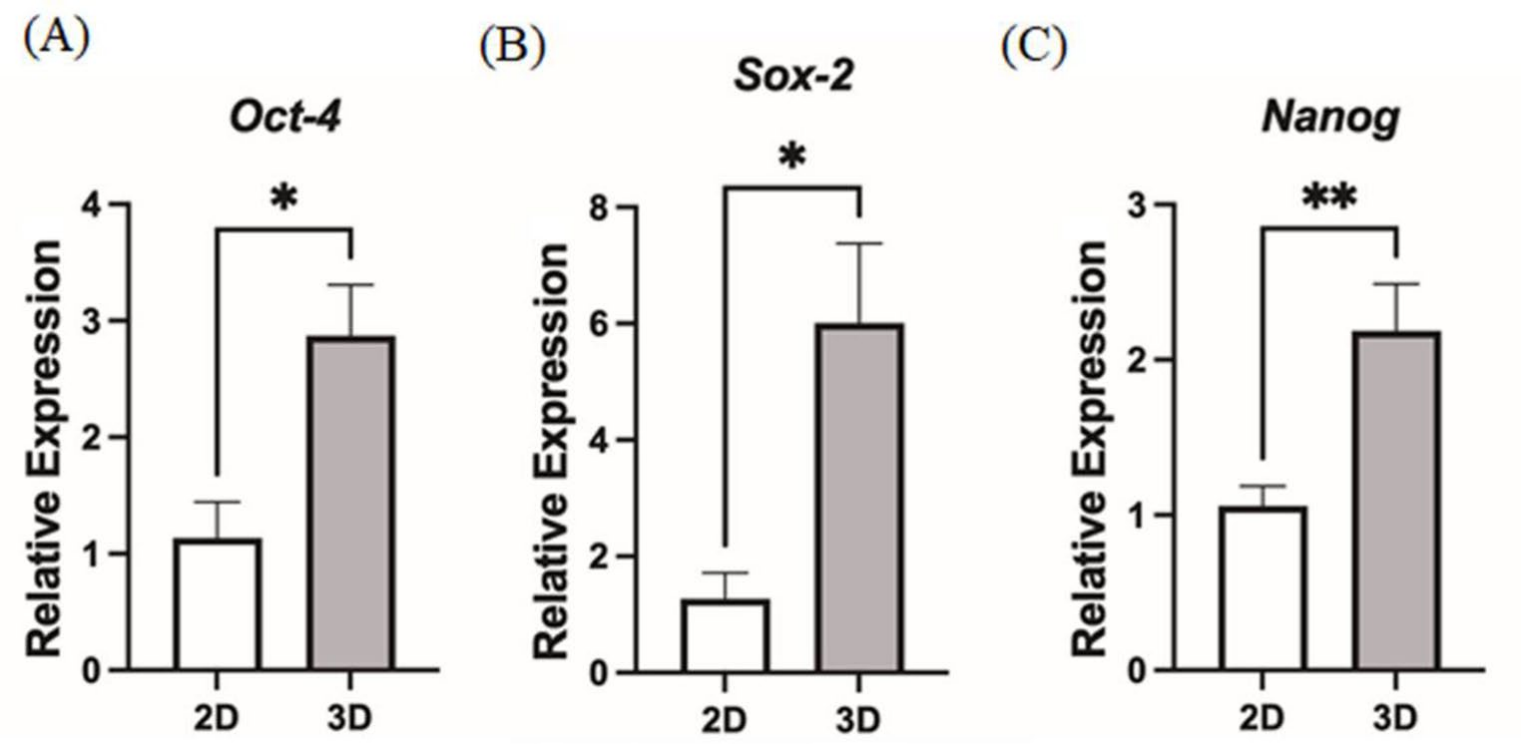
The pluripotent genes were upregulated in MSC in 3D culture. To examine whether the stemness of the MSCs was affected after being cultured in hanging drop for 48 h. The expression of pluripotent genes (**A**) *Oct4*, (**B**) *Sox2*, and (**C**) *Nanog* were determined by RT-qPCR. (*n* = 3, Each column represents the mean ± standard error of the mean of at three independent experiments. *p < 0.05, **p < 0.01)

We next investigated the influence of the stemness characteristics of MSCs by 3D culture. hMSCs were placed in a hanging drop for 48 h, and then the cells were collected and subjected to real-time PCR for analyzing the expression of critical pluripotency genes, such as Oct4, Sox2, and Nanog. Real-time PCR data suggested upregulation of Oct4, Sox2, and Nanog from 3D culture compared to 2D culture (Figs. 6A-C). It demonstrated that the 3D culture might have upregulated the expression of core stemness markers, thereby creating a markedly more primitive therapeutic MSC phenotype.

## Discussion

The therapeutic potentials of 3D cultured MSCs have been previously reported. When grown as three-dimensional spheroids, MSCs exhibit enhanced angiogenic, anti-inflammatory, and immunomodulatory properties, along with better stemness and survival rates following transplantation. While these functional improvements are well documented, the molecular mechanisms underlying these enhancements have not been comprehensively elucidated. For example, the short-term 3D culture of the MSCs may have the ideal microenvironment to enhance their regenerative capacity, which needs thorough elucidation of the mechanisms involved. In this study, we reported a series of cellular alterations induced by 3D culture, including decreased proliferation and cell cycle arrest, mitigation of lung or tissue trap when administrated intravenously, enhancement of cell signaling to extrinsic signals, reduction of cell-matrix binding, and upregulation of stemness genes. These findings reveal extensive phenotypic remodeling and global transcriptomic reprogramming in 3D MSCs. Lu and colleagues have recently discovered that MSC spheroids prepared using spinner flasks showed upregulated gene expression in bulk RNA analysis after 3D culture, particularly in immunosuppressive factors and growth factors-related genes [30]. However, our comparative transcriptome data identified the upregulation of cellular responses to immunological and extracellular responses, extracellular matrix organization and vasculature development, and downregulation of cell-cell or cell-matrix binding and actin organization as the results of 3D culture. Such transcriptomic shifts align closely with the observed functional changes in MSC migration, tissue engraftment, and regenerative potential. Critically, our study combines phenotypic analysis and transcriptomic profiling to reveal novel mechanisms by which 3D culture alters the therapeutic properties of MSCs, contrasting with previous studies that focused exclusively on functional outcomes. Together, these findings suggest that the 3D hanging drop culture renders MSCs favorably modified in multiple functional aspects, yet it is also a viable path to advance MSCs clinical applications in regenerative medicine.

MSCs are attracted to various chemokines and growth factors, such as tumor necrosis factor-α (TNF-α), interleukin-6 (IL-6), interferon-γ (IFN-γ), stromal-derived factor-1 (SDF-1), and monocyte chemoattractant protein-1 (MCP-1). Additionally, these cells possess a range of surface receptors, including VCAM-1, CXC motif, and CD44, which facilitate the expression of various chemokines and growth factors, including PDGF (platelet-derived growth factor), HGF (hepatocyte growth factor), VEGF (vascular endothelial growth factor), and bFGF (basic fibroblast growth factor) [36, 37]. Additionally, these cells undergo downstream signaling pathway activation [36]. Aligned with the findings above, we also found considerable enrichment of chemokine signaling pathways in our 3D MSC transcriptomic data analysis. Upregulation of chemokine receptor genes, such as CXCR4 and CCR2, has been identified in cells derived from MSC spheroids. The directional cue is, moreover, reinforced by sensitizing hMSCs to other chemokines like SDF-1 [38], that work as chemoattractants for multiple stem cell types, wherein MSCs, hematopoietic stem cells, neural stem cells, and endothelial progenitor cells [39]. The data from the chemotaxis assays showed that 3D MSCs from the spheroid culture displayed significantly greater chemotactic migration compared to their 2D counterparts. Implying that the 3D culture niche enhances chemotactic responsiveness rather than enhancing motility. This enhancement might be perceived at the functional level through chemokine receptor stimulation, for example, CXCR4, CCR2, and TNFRSF1B. Indeed, the overexpression of these receptors implies that the 3D MSCs may have an enhanced homing ability to tumors and tissue-damaged regions.

Lung entrapment remains a significant obstacle for clinical applications of intravenous administration of MSCs. This phenomenon reduces the homing effects of MSCs and increases the risk of pulmonary embolism as MSCs are entrapped in lung microvasculature. Prior research has indicated that 3D spheroid culture reduces lung retention, possibly because of a smaller cell size. Our in vivo data also confirmed it since animals receiving 2D MSCs have shown increased pulmonary accumulation and higher occurrence of respiratory distress compared to those receiving 3D MSCs. For instance, several animals injected with 2D MSCs showed initial symptoms of shortness of breath just hours after injection compared to those with no symptoms in the same time frame receiving 3D MSCs. Nevertheless, our comparative transcriptomics implied that this reduced lung entrapment may not merely result from the reduced cell size of 3D MSCs. Indeed, significant downregulation in pathways related to focal adhesion, integrin signaling, and actin cytoskeleton organization has been observed in the 3D MSCs. With reduced expression of adhesion molecules and cytoskeletal rigidity, MSCs may get deformed easily and pass through narrow capillaries resulting in less lung entrapment.

There are a few disadvantages associated with 3D spheroid culture for their widespread clinical use [9, 40]. The first is the relatively low recovery rates of MSCs. Static-formed spheroids are constructed of three zones: outer layer, middle region, and inner core. Due to the compactness of the structure, cellular compressibility increases toward the center, while nutrient and oxygen diffusion progressively declines. Concurrently, the cells’ metabolic activity and proliferative capacity diminish in the deeper layer. These nutrient and oxygen gradient factors further impact the heterogeneity in cell viability within the layers. The peripheral region contains MSCs with intact and normal nuclei, performing asynchronous robust proliferation and metabolism. The middle region accommodates MSCs with condensed nuclei showing sustained proliferation and metabolism. The inner region showed cells with fragmented and senescent nuclei, forming the dead core zone of the spheroid. As a result, only cells in the outer layer have the optimal conditions for therapeutic use. Moreover, proliferation assays and cell cycle gene analysis revealed that MSC proliferation was impeded due to 3D spheroid culture, although some proliferative potential remained. Therefore, modifications in culture systems need to be adopted to enhance the quality and quantity of 3D MSCs for clinical applications. Alternative strategies, such as increasing the diffusion of nutrients and oxygen through dynamic culture systems or perfusion-based 3D bioreactors, may help counteract the issues associated with static spheroid culture.

## Data Availability

All data generated or analyzed during this study are included in this published article, which are available from the corresponding author on reasonable request.
